# Sleep Quality and Its Relationship to Anxiety and Hardiness in a Cohort of Frontline Italian Nurses during the First Wave of the COVID-19 Pandemic

**DOI:** 10.3390/nursrep13030103

**Published:** 2023-09-07

**Authors:** Samuele Baldassini Rodriguez, Yari Bardacci, Khadija El Aoufy, Marco Bazzini, Christian Caruso, Gian Domenico Giusti, Andrea Mezzetti, Alberto Lucchini, Pasquale Iozzo, Andrea Guazzini, Camilla Elena Magi, Paolo Iovino, Yari Longobucco, Laura Rasero, Stefano Bambi

**Affiliations:** 1 Emergency and Trauma Intensive Care Unit, Careggi University Hospital, 50134 Florence, Italy; samuelebr@hotmail.com (S.B.R.); bardacci.yari@gmail.com (Y.B.); bazzinim@aou-careggi.toscana.it (M.B.); 2 Department of Experimental and Clinical Medicine, University of Florence, 50134 Florence, Italy; 3 Emergency Medical System—AUSL Toscana Centro, 50122 Florence, Italy; christian.caruso@uslcentro.toscana.it (C.C.); andrea@mezzetti.it (A.M.); 4 Medicine and Surgery Department, University of Perugia, 06100 Perugia, Italy; giustigiando@gmail.com; 5 Teaching and Quality Department, Perugia University Hospital, 06100 Perugia, Italy; 6 UOS Terapia Intensiva Generale e UOSD Emergenza Intraospedaliera e Trauma Team, Fondazione IRCCS San Gerardo dei Tintori, 20900 Monza, Italy; alberto.lucchini@unimib.it; 7 Emergency Department, Azienda Ospedaliera Universitaria Policlinico Paolo Giaccone, 90100 Palermo, Italy; pasqualeiozzo72@gmail.com; 8 Department of Education, Languages, Intercultural Studies, Literatures and Psychology, University of Florence, 50135 Florence, Italy; andrea.guazzini@gmail.com; 9 Center for the Study of Complex Dynamics (CSDC), University of Florence, 50134 Florence, Italy; 10 Department of Health Sciences, University of Florence, 50134 Florence, Italy; camillaelena.magi@unifi.it (C.E.M.); paolo.iovino@unifi.it (P.I.); yari.longobucco@unifi.it (Y.L.); l.rasero@unifi.it (L.R.); stefano.bambi@unifi.it (S.B.)

**Keywords:** insomnia, healthcare workers, healthcare professionals, nurses, hardiness, COVID-19, pandemic

## Abstract

Introduction: The COVID-19 pandemic has had a considerable impact on the psychological and psychopathological status of the population and health care workers in terms of insomnia, anxiety, depression, and post-traumatic stress disorder. The primary aim of this study was to describe and evaluate the impact of the pandemic on insomnia levels of a cohort of Italian nurses, particularly those involved in the care of COVID-19 patients. The secondary aim was to identify the interaction between insomnia and hardiness, anxiety, and sleep disturbances. Materials and Methods: A descriptive–exploratory study was conducted using an online survey during the first wave of the COVID-19 pandemic (March to July 2020). The questionnaire consisted of multiple-choice, open-ended, closed, and semi-closed questions. The psychometric tools administered were the Dispositional Resilience Scale (DRS-15), the State–Trait Anxiety Inventory (STAI-Y), and the Insomnia Severity Index (ISI). Results: a cohort of 1167 nurses fully completed the questionnaire (86.2% of total respondents). The insomnia scale survey showed an increase in post-pandemic scores compared to those before the pandemic, implying that insomnia levels increased after the first pandemic wave. Insomnia scores were directly correlated with anxiety levels (r = 0.571; *p* ≤ 0.05) and inversely correlated with hardiness levels (r = −0.324; *p* < 0.001). Multivariate analysis revealed the following protective factors: not having worked in COVID-19 wards, high levels of hardiness (commitment), and the presence of high pre-pandemic insomnia disorder. The main risk factor for insomnia reported in the analysis was a high anxiety score. Discussion and Conclusion: Anxiety represented the main risk factor for insomnia severity in our sample, while hardiness was confirmed as a protective factor. Thus, it is necessary to design further studies to identify additional risk factors for poor sleep quality and to develop educational courses and strategies aimed at enhancing rest and sleep quality, especially for frontline nurses.

## 1. Introduction

Sleep and rest are fundamental physiological needs of humans. Indeed, adequate sleep provides the recovery and maintenance of energy, efficiently improves physical and intellectual functions, and promotes well-being and emotional balance [1]. Nurses, among all healthcare professionals, are particularly exposed to sleep disturbances [2] and insomnia for the changes in sleep–wake rhythm due to the variation in working hours, covering 24 h shifts (namely morning, afternoon, and night shifts) [3,4], heavy workloads, the emotional and psychological impact that some clinical settings can exert [5], and the occurrence of burnout [6,7], as well as the physiological process of decreased sleep hours, not to mention increased sleep fragmentation due to aging [8].

According to a recent meta-analysis, the prevalence of sleep disturbances in Chinese nurses was 45.1% (CI 95%: 37.2–53.1%) [9], while Ielapi et al. reported a prevalence of 65.4% [10]. Sleep disturbances can influence personal well-being and mental health, also causing depression [11], which has been reported in 60.3% of cases [12]. Furthermore, observational research has shown that a reduction in the time dedicated to sleep is associated with decreased productivity [13], quality of care, and patient safety [14]. These features took on particularly important aspects during the first waves of COVID-19 pandemic. Pooled data from 44 meta-analyses showed that the prevalence of sleep disturbance reported among hospital healthcare workers during the first waves of the COVID-19 pandemic was 40% (CI 95%: 36.9% to 42.0%) [15]. Moreover, in a meta-analysis published in 2020, nurses accounted for 34.8% of sleep disturbance (CI 95%: 24.8–46.4%) [16]. The literature also reports the deterioration of healthcare workers’ psychological and emotional status due to anxiety, fear, depression, and fatigue [17,18,19,20].

In addition to the worsening of sleep quality, other psychological conditions negatively affected nurses operating in COVID-19 hospital areas, namely a pooled prevalence of anxiety at 16–41%, depression at 14–37%, and stress/post-traumatic stress disorder at 18.6–56.5% were reported in a recent meta-review of systematic reviews about the mental health status of healthcare professionals during the COVID-19 pandemic [21].

Anxiety was reported in percentages varying from 20% to 72% among healthcare professionals working in first-line COVID-19 settings in many countries [22]. Moreover, anxiety has been shown to affect nurses’ ability to relax and obtain adequate quality of sleep, thus presenting a significant risk factor for insomnia [23,24]. During the first wave of COVID-19, high levels of anxiety were registered among all healthcare professionals, especially nurses, due not only to the novelty of the SARS-CoV2 infective threat but also to the lack of personal protective equipment (PPE) and training, fatigue due to the wearing of PPE for many consecutive hours, and the absence of a vaccine that could have protected first-line operators [25,26,27].

However, the way nurses faced this global emergency showed the presence of psychological points of strength, identified in the concept of resilience [28], which was positively related to the quality of general life and working life [29,30] and showed negative correlations with depression and burnout while mitigating the effect of pandemic fatigue on mental health and sleep quality [31,32,33,34].

When dealing with resilience, we should remember that it has a fundamental antecedent, represented by hardiness, a multifaceted behavioral approach characterized by three components: (i) commitment: the motivation to engage fully in one’s work and personal life, even in the face of adversity; (ii) control: the belief that one can influence outcomes via one’s actions and efforts; and (iii) challenge: the view that change and adversity are opportunities for growth and development [35,36,37,38]. This represents a very important behavioral trait, as adequate levels of hardiness allow individuals to face challenging and dangerous situations by showing the ability to counterbalance the negative emotional and psychological effects of catastrophic conditions and to be prepared to face life challenges. Previous studies have shown a relationship between hardiness levels and anxiety in nurses employed as first-line healthcare providers during the first wave of the COVID-19 pandemic [39]. However, to date, scientific literature has not analyzed the influence of hardiness on insomnia levels in nurses. Therefore, we designed a study to identify the relationship between insomnia, anxiety, and hardiness in a cohort of nurses working in hospital and out-of-hospital clinical settings during the first wave of the COVID-19 pandemic. Therefore, the primary aim of this study was to describe the insomnia levels of Italian nurses during the first wave of the COVID-19 pandemic, specifically those involved in the care of COVID-19 patients. The secondary aim was to determine the effect of hardiness and anxiety levels on the occurrence of insomnia as well as the associated risk and promoting factors.

## 2. Materials and Methods

The study has been conducted consistently with the STROBE for observational studies [40].

### 2.1. Study Design

During the first wave of the COVID-19 pandemic, a descriptive exploratory study was conducted by developing (March to April 2020) and disseminating (May to July 2020) an online survey. 

### 2.2. Participants

All healthcare workers in Italy who were involved in the care of COVID-19 patients during the first wave of the pandemic and those who cared for non-COVID-19 patients made up the sample for the original Anxiety–Insomnia–Resiliency COVID-19 Study (AIR-COVID-19). The inclusion criteria were as follows: all healthcare workers with an unlimited or fixed-term job contract; acceptance; and signature of informed consent for study participation, aiming to include as many participants as feasible as no sample size calculation was made. However, only the nurses’ responses were considered in this descriptive study.

### 2.3. Methods

The Italian Association of Critical Care Nurses (ANIARTI) provided the Survey Monkey online platform, which was used to conduct this web survey. A link to the anonymous questionnaire completion process was made available through the websites and social media accounts of healthcare professional organizations. Following the first wave of the pandemic in Italy, the survey period began in May 2020 and lasted for 60 days. Approximately 10–12 min were needed to complete the survey, and participants were free to leave the study at any point.

### 2.4. Outcome Measures

The survey consisted of open, closed, and semi-closed-ended multiple-choice items. The answers to the closed questions might take many different forms, such as multiple, dichotomous, or rating (using a Likert scale) responses. In particular, participants were asked about socio-demographic data (sex, age, marital status, number of children, level of education, and profession), and some of the questions were intended to gather information on participants’ healthcare settings, their involvement in the care of COVID-19 patients, their relocation due to the pandemic emergency, and the distance between their home and their present place of employment.

The second section of the survey was composed of Italian versions of three psychometric instruments: the Dispositional Resilience Scale (DRS-15) [36], the State–Trait Anxiety Inventory (STAI-Y) [41], and the Insomnia Severity Index [42].

The Dispositional Resilience Scale (DRS-15), which was used to measure hardiness, is a valid, reliable, and concise psychometric tool for the self-assessment of hardiness. This scale assesses degree of psychological resistance or overall functioning style, which includes cognitive, emotional, and behavioral characteristics. The original version comprised 45 items with acceptable psychometric properties [36,43,44]. The Italian version of the DRS-15 shows good levels of reliability and stability (Cronbach α of 0.73; Intra-Class Correlation of 0.75 between two administrations after a time interval of one month) and evidence of construct and criterion validity. It consists of three dimensions (subscales): “Commitment”, “Control”, and “Challenge”. Each item asks the participant to state the level of truth about a single affirmation on a 4-point Likert scale (from “1—not at all true” to “4—completely true”).

The State Trait Anxiety Inventory (STAI-Y) was used to assess anxiety levels; composed of 40 items, the questionnaire measures both state and trait anxiety using a 1–4 point Likert rating scale (from “1—none” to “4—severe”) [41,45]. On the STAI-Y1 (items 1–20), the intensity of feelings “in this moment” was assessed (Cronbach α in adults 0.91), while on the STAI-Y2 (items 21–40), the focus was on the frequency of feelings “in general” (Cronbach α in adults 0.85). However, in our study, we considered only state anxiety, as it is more sensitive than trait anxiety and highly related to trait anxiety (0.8) [46]. The score ranged between 20 and 80; the anxiety cut-off value was 40, where a score higher than 60 indicated severe anxiety.

The Insomnia Severity Index (ISI) is a 7-item self-report questionnaire assessing the nature, severity, and impact of insomnia via a 5-point Likert scale during the “last month”, yielding a total score ranging from 0 to 28 [47]. The total score was interpreted as follows: absence of insomnia (0–7); sub-threshold insomnia (8–14); moderate insomnia (15–21); and severe insomnia (22–28). The dimensions evaluated were severity of sleep onset, sleep maintenance, early morning awakening problems, sleep dissatisfaction, interference of sleep difficulties with daytime functioning, noticeability of sleep problems by others, and distress caused by sleep difficulties. Linguistic validation of the scale is provided in many European and non-European languages, and the scale has been proven to retain good psychometric properties in the translated versions [48]; indeed, the Italian version of the ISI is a valid and reliable instrument (internal reliability coefficient −0.75) for the assessment of subjective symptoms of insomnia and is frequently used in both research and clinical practice [42].

### 2.5. Ethical Considerations

The local Ethical Committee in the Tuscany Region (Italy) at the time of the study implementation did not cover the approval of observational studies performed on healthcare workers, but nevertheless recommended the use of informed consent and protection of personal data in accordance with the current national privacy legislation. We followed both these indications during the design and performance of the present study. This is the reason why we did not seek any ethical approval before the beginning of this study. Thus, the study protocol was designed in accordance with GCP (Good Clinical Practice) and was conducted in compliance with the Helsinki Declaration. Additionally, this research was performed in accordance with the principles of the Body of Privacy Law (Italian legislation numbers 196/2003 and 101/2018). An individual sequential code number was issued to each participant’s data, all of which were then gathered and handled to protect anonymity. The findings were uploaded to an .xls file, which was accessible only to the researchers and password-protected.

### 2.6. Data Analysis

The data analysis process was divided into four stages. In the first stage, we preprocessed, codified, and cleaned the datasets from the survey, discretizing and changing the metrics of the observables whenever the conditional balancing did not meet the requirements for the subsequent inferential analysis. The IBM Statistical Package for Social Sciences (SPSS 27.0) was used to conduct studies on the frequencies, central tendencies, and dispersion indicators as part of the second stage [49]. Then, the normality of the distribution of the continuous variables was verified by assessing whether the asymmetry and kurtosis values fell in the interval between −1 and +1, as well as a sufficient balance and size, before moving on to the inferential analyses. In the third and last stages, we investigated the univariate relations between the selected observables using the Pearson r correlation to compare continuous variables and repeated measures ANOVA to evaluate the impact of dichotomous observables on continuous ones and in particular the effects of time (i.e., pre–post pandemic first wave effect) on insomnia.

## 3. Results

A cohort of 1693 healthcare providers, of which 1354 were nurses, were included in the original AIR-COVID-19 study. In the current study, 1167 nurses filled out the questionnaire regarding insomnia, thus maintaining 86.2% (1167/1354 nurses) of respondents (81.2 % women—4948/1167), with a mean age of 42.4 (SD ± 10.7) years (CI 95% 41.7–43.0) and a mean length of service of 17.9 ± 11.6 years (CI 95% 17.2–18.6).

As a consequence of the COVID-19 pandemic, nurses were reallocated to another unit in 27.8% (324/1167) of cases, and 33.6% (109/324) were transferred to a COVID-19 unit. Transferees reported an average positive satisfaction rate of 78.4%. Moreover, 383 out of 1167 (32.8%) nurses reported caring for COVID-19 patients (784/1167 did not). The perception of being adequately provided with personal protective equipment (PPE) was also assessed, and 52.1% (608/1167) reported satisfaction. Results reported in Table 1 show that nurses who served in COVID-19 wards were significantly younger (40.9 ± 10.3 vs. 43.1 ± 10.8; *p* < 0.001) and with lower seniority (16.6 ± 11.3 vs. 18.5 ± 11.7; *p* < 0.01) when compared to nurses not involved in caring for COVID-19 patients. As for other characteristics, such as the transfer of department, its evaluation, and the perception of being provided with adequate PPE, no statistically significant differences between the two groups were reported (Table 1).

As for the state and trait anxiety values, the nurses enrolled in the study reported values of 47.0 ± 12.4 and 42.6 ± 10.1, respectively, meaning that both the state (48.8 ± 12.5 vs. 46.1 ± 12.2; *p* < 0.001) and trait (43.5 ± 10.0 vs. 42.2 ± 10.2; *p* < 0.05) anxiety levels were significantly higher in the group of nurses caring for COVID-19 patients (Table 2).

As for insomnia levels, the descriptive results in Table 3 show that similar percentages reported no clinically significant insomnia in either group (57.5% nurses caring for COVID-19 patients and 58.8% of those who did not) before the beginning of the pandemic, while after the first wave, sub-threshold and clinical insomnia were more represented, showing a shift of respondents from the first group before the beginning of the pandemic to the other three groups after the first wave of the pandemic (Table 3).

Moreover, pre-score insomnia levels were lower than post-score levels, meaning that insomnia levels worsened after the first COVID-19 pandemic wave in the whole sample and in both groups of nurses. However, our data showed significant differences between the two groups of nurses in post- and delta insomnia scores (Table 4).

Hardiness levels (measured by DRS total) showed lower values after the first wave of the pandemic than before the beginning of the pandemic for both groups of nurses, as reported in Table 4. No statistically significant differences in hardiness levels (total, control, challenge, and commitment) assessed before the beginning of the pandemic were reported in the groups of nurses, whereas statistically significant differences between the groups were reported for total hardiness and challenge levels assessed after the first wave of the pandemic (*p* < 0.05).

Regarding hardiness delta levels (post–pre), our data showed negative scores for the hardiness total (−1.3 ± 5.1; CI 95% −1.6; −1.0; *p* < 0.01) and all subscales, indicating that hardiness levels decreased after the first wave of the pandemic, and all the scores showed statistically significant differences between the two groups of nurses (*p* < 0.05).

Regarding the inferential analysis assessing the correlation between insomnia levels and other variables, no significant differences were found in relation to gender, age, or length of service in the entire sample of nurses after the first wave of the COVID-19 pandemic. However, insomnia levels were directly and significantly correlated with anxiety trait–state levels (*p* ≤ 0.05) and inversely and significantly correlated with hardiness levels (total and subscales) (*p* ≤ 0.05) (Table 5).

**Figure 1:**
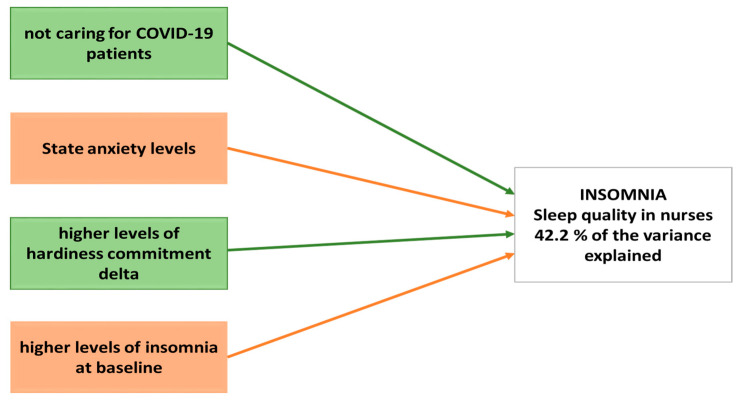
Representation of the best predictive model for insomnia levels.

Finally, a generalized linear model was used to calculate the best predictive model for insomnia levels among nurses during the first wave of the COVID-19 pandemic. Our results showed that 42.2% of the variance was explained by four variables: (i) not caring for COVID-19 patients, (ii) higher levels of hardiness commitment delta, (iii) state anxiety, and (iv) higher levels of insomnia at baseline. Indeed, the general linear model takes the original level of insomnia into account, clearly demonstrating that greater initial insomnia results in a lower possibility of symptom progression or deterioration (Table 6 and Figure 1). 

## 4. Discussion

Our results show that insomnia levels referred to by the participants before the beginning of the pandemic were above the average of general population; however, insomnia levels increased further after the first wave of the pandemic. Moreover, anxiety levels also increased due to the pandemic and were directly and significantly associated with insomnia levels. In particular, our results show a statistically significant difference, not only before and after the first wave of the pandemic, but also in the two subgroups considered, i.e., those nurses who were caring for COVID-19 patients and those who were not. Finally, dispositional resilience plays a pivotal role in the final score of insomnia levels, depending on low scores (risk factors) or high scores (promoting factor).

In our previous study, we analyzed the promotive and risk factors of hardiness levels in nurses involved in the care of COVID-19 patients, showing that length of service, positive evaluation of department reallocation, and, surprisingly, inadequate PPE when considering a positive assessment of department reallocation constituted promotive factors [39]. The risk factors for the worsening of hardiness levels were anxiety alone, the association of anxiety with length of service, the negative assessment of department reallocation, and the evaluation of insufficient PPE when associated with a negative assessment of department reallocation [39]. Thus, in the present study, we focused on the insomnia and sleep quality of nurses, and any differences were revealed between the beginning of the pandemic and after the first wave of the pandemic.

Several studies on mental distress in healthcare workers were conducted during the first wave of the COVID-19 pandemic (from March to May 2020); however, studies carried out during subsequent waves showed similar outcomes, either demonstrating that different regions were hit harder by the pandemic during different periods or highlighting the persistence of the pandemic and the subsequent impact on nurses’ mental health status [16]. Likewise, in accordance with our previous results on hardiness, a systematic review by García-Vivar et al. highlighted the mental health effects on nurses working in different parts of the world during the COVID-19 pandemic [50]. Indeed, the authors claim that nurses, among all healthcare workers, reported the highest levels of psychological distress because of their working conditions, which worsened when they were female and lacked access to PPE [50]. Accordingly, in our study, the group of nurses caring for COVID-19 patients reported significantly higher values for state and trait anxiety levels than those who did not, indicating that the impact of the COVID-19 pandemic played an important role in nurses’ anxiety. In addition, insomnia levels were directly correlated with anxiety trait levels.

Moreover, a study conducted in Italy by Simonetti et al. (2021) reported the worst outcomes compared with similar studies included in the systematic review by García-Vivar et al., with 75.72% of nurses reporting poor sleep quality (data collected from February to April 2020) [50,51]. Accordingly, all the included studies examining sleep quality described overall negative outcomes and a high prevalence of sleep disturbances in nurses. In addition, independent of the COVID-19 pandemic, nurses are at a high risk of insomnia, as reported in a systematic review by Booker et al. (2018) [52]. Indeed, our results on insomnia levels showed similar baseline percentages in both groups (57.5% nurses caring for COVID-19 patients and 58.8% of nurses not caring for COVID-19 patients), with a shift towards sub-threshold and clinical insomnia immediately after the first wave of the pandemic, highlighting the worsening of sleep quality and disturbances (as shown in Table 3). Liu et al. (2020) [53] in their cross-sectional study reported that the percentage of medical staff who suffered from insomnia in China was 32.0%, lower than similar previous studies but higher than the 30.5% prevalence of insomnia in non-medical personnel under the COVID-19 epidemic [54]. Italian nurses participating in the study reported higher ISI scores (more than 55%) for insomnia, which is likely due to the fact that at the start of the COVID-19 outbreak, Italy was the first European country affected, and due to the unpreparedness of the national health system, medical staff lacked PPE and knowledge of the disease, which increased their anxiety, fear, and insomnia.

Furthermore, in our sample, pre-score insomnia levels were lower than post-score levels, indicating that insomnia levels worsened after the first COVID-19 pandemic wave in the whole sample of nurses, with statistically significant differences between the two groups in the post- and delta scores (as shown in Table 4); that is, nurses caring for COVID-19 patients reported worse levels. Surprisingly, Nashwan et al., in their cross sectional study on 200 nurses in Qatar, reported no statistically significant differences between COVID-19 and non-COVID-19 facilities for insomnia levels; we assume that these results could be influenced and explained either by the nursing management support of nurses or the rapid adaptation to the pandemic condition [55].

Moreover, despite the number of published papers to date, only one study has dealt with the correlation between psychological hardiness and insomnia in nurses during the COVID-19 pandemic [56]. However, hardiness [57] is a critical trait for nurses, who face several challenges and stressors during their work shifts. Nurses who show higher levels of hardiness are better equipped to cope with these challenges while maintaining their well-being.

As for hardiness levels, lower values were reported after the first wave of the COVID-19 pandemic for both groups of nurses, with statistically significant differences between the groups only for total hardiness and challenge. Similarly, hardiness levels decreased after the first wave of the pandemic, and all scores showed statistically significant differences between the two groups of nurses. Insomnia levels were inversely correlated with hardiness levels.

When dealing with sleep quality, we should remind the reader that an important relationship between mental health distress, namely sleep quality, and burnout has previously been demonstrated in nurses [6]. Indeed, a recent systematic review and meta-analysis suggested that a considerable proportion of healthcare workers experienced mood and sleep disturbances, especially due to the pandemic, stressing the need to establish ways to mitigate mental health risks and deliver appropriate interventions [56]. In fact, high work pressure and uncertainty about the risks of COVID-19 increased nurses’ anxiety, depression, post-traumatic stress disorder [58], emotional exhaustion [59], and burnout [32] rates.

Considering this, our study confirms that hardiness represents one of the most important factors to consider and implement as a risk factor (when displaying low values) or a promotive factor (when displaying high values) for insomnia and sleep quality [59].

Thus, a predictive model was developed using a generalized linear model that showed 42.2% of the variance was explained by four variables: not caring for COVID-19 patients, higher levels of hardiness commitment delta, state anxiety, and higher levels of insomnia at baseline. Clearly, the baseline insomnia score is taken into account, providing validity to greater initial insomnia, resulting in the possibility of symptom progression or deterioration; thus, strategies based on sleep hygiene should always be implemented to enhance sleep quality at all times, not only in the case of catastrophic events. However, the high pre-pandemic insomnia level as a protective factor can be counterintuitive and might merit further investigation to confirm this conclusion.

Our study is not without limitations; in fact, we are well aware that the online survey performed after the first wave of the pandemic in Italy questioned our respondents about how they felt before the beginning of the pandemic: this aspect may be affected by a recall bias, as the data are based on respondents’ memory and experience. We cannot exclude the presence of a self-selection bias, which is typical in survey design research.

In conclusion, the assessment of hardiness as a behavioral trait in healthcare professionals, especially nurses, might be useful to identify individuals with low or high scores and optimize the allocation of human resources by nurse managers. The experience of the COVID-19 pandemic has taught us that it is mandatory to prioritize healthcare professionals’ wellbeing during high-stress periods due to hard working conditions and high workload. Thus, it is necessary to design further studies to identify additional risk factors for poor sleep quality and to develop educational courses and strategies aimed at enhancing rest and sleep quality, especially for frontline nurses.

## Figures and Tables

**Table 1:** Correlations between the two groups of nurses (i.e., those involved in the care for COVID-19 patients and those who were not involved) and sociodemographic or other variables.

Dimension	μ/f	σ/%	CI 95%	Welch t/χ^2^
Gender				
Total Sample	1167	81.2% (F)		
Sample COVID Yes	383	76.8% (F)		7.48 **
Sample COVID No	784	83.4% (F)		
Age				
Total Sample	42.4	10.7	41.7–43.0	
Sample COVID Yes	40.9	10.3	39.9–42.0	3.29 ***
Sample COVID No	43.1	10.8	42.3–43.8	
Length of service				
Total Sample	17.9	11.6	17.2–18.6	
Sample COVID Yes	16.6	11.3	15.5–17.7	2.74 **
Sample COVID No	18.5	11.7	17.7–19.4	
Ward/ department transfer				
Total Sample	324/1167	27.8%		
Sample COVID Yes	109/383	28.5%		0.14
Sample COVID No	215/784	27.4%		
Satisfaction levels about ward/department transfer				
Total Sample of transferees	254/324	78.4%		
Sample COVID Yes	84/109	77.1%		0.17
Sample COVID No	170/215	79.1%		
Perception of adequate PPE				
Total Sample	608/1167	52.1%		
Sample COVID Yes	198/383	51.7%		0.04
Sample COVID No	410/784	52.3%		

μ: Average value; σ: Standard deviation; f: frequency; %: Percentage (x); C.I.: Confidence Interval; ***: *p* < 0.001; **: *p* < 0.01.

**Table 2:** Descriptive analysis and Welch test/χ^2^ to assess the correlation between the two groups of nurses (i.e., those involved in the care for COVID-19 patients and those who were not involved) and trait–state anxiety levels.

Dimension	μ	σ	CI 95%	Welch t/χ^2^
STAI—Y2 (state)				
Total Sample	47.0	12.4	46.3–47.7	
Sample COVID Yes	48.8	12.5	47.6–50.1	−3.57 ***
Sample COVID No	46.1	12.2	45.2–46.9	
STAI—Y1 (trait)				
Total Sample	42.6	10.1	42.0–43.2	
Sample COVID Yes	43.5	10.0	42.5–44.5	−1.96 *
Sample COVID No	42.2	10.2	41.5–42.9	

μ: Average value; σ: Standard deviation; C.I.: Confidence interval; ***: *p* < 0.001 *: *p* < 0.05.

**Table 3:** Insomnia severity as assessed via ISI in the two sub-samples of nurses.

	COVID Yes (383)		COVID No (784)	
Insomnia Severity Index	Pre N (%)	Post N (%)	Pre N (%)	Post N (%)
No clinically significant insomnia	220 (57.5)	85 (22.2)	461 (58.8)	229 (29.2)
Sub-threshold insomnia	133 (34.7)	156 (40.7)	265 (33.8)	331 (42.2)
Clinical insomnia (moderate severity)	30 (7.8)	115 (30)	52 (6.6)	183 (23.4)
Clinical insomnia (severe)	0 (0)	27 (7.1)	6 (0.8)	41 (5.2)

**Table 4:** Descriptive analysis and Welch test/χ^2^ to assess the correlation between the two subgroups of nurses (i.e., involved in caring for COVID-19 patients and those who were not involved) and insomnia and hardiness sub-scores.

Dimension	*Pre*			*Post*			*Delta* ^1^		
	μ(σ)	CI 95%	Welch t/χ^2^	μ(σ)	CI 95%	Welch t/χ^2^	μ(σ)	CI 95%	Welch t/χ^2^
DRS Total									
Total Sample	28.0 (5.4)	27.7–28.3		26.7 (6.7)	26.3–27.1		−1.3 (5.1)	−1.6; −1.0	
Sample COVID Yes	28.0 (5.2)	27.5; 28.5	0.14	26.0 (7.0)	25.3; 26.7	2.30 *	−1.9 (5.4)	−2.5; −1.4	2.84 **
Sample COVID No	28.0 (5.5)	27.6; 28.4		27.0 (6.6)	26.6; 27.5		−1.0 (4.9)	−1.4; −0.7	
DRS Commitment									
Total Sample	10.1 (2.4)	10.0–10.3		9.3 (3.0)	9.1–9.4		−0.9 (2.3)	−1.0; −0.7	
Sample COVID Yes	10.1 (2.3)	9.9; 10.4	0.15	9.1 (3.1)	8.7; 9.4	1.68	−1.1 (2.5)	−1.3; −0.8	1.97 *
Sample COVID No	10.1 (2.4)	10.0; 10.3		9.4 (3.0)	9.2; 9.6		−0.8 (2.2)	-0,9; −0.6	
DRS Challenge									
Total Sample	8.4 (3.0)	8.2–8.6		8.2 (3.2)	8.0–8.4		−0.2 (2.1)	−0.3; −0.1	
Sample COVID Yes	8.3 (3.0)	8.0; 8.6	0.69	7.9 (3.3)	7.5; 8.2	2.46 **	−0.5 (2.1)	−0.7; −0.2	2.86 **
Sample COVID No	8.5 (3.0)	8.2; 8.7		8.4 (3.2)	8.1; 8.6		−0.1 (2.1)	−0.2; 0.1	
DRS Control									
Total Sample	9.4 (2.2)	9.3–9.6		9.2 (2.4)	9.1–9.3		−0.2 (2.0)	−0.4; −0.1	
Sample COVID Yes	9.5 (2.0)	9.3; 9.7	−0.79	9.1 (2.4)	8.9; 9.3	1.04	−0.4 (2.0)	−0.6; −0.2	2.14 *
Sample COVID No	9.4 (2.2)	9.3; 9.6		9.3 (2.4)	9.1; 9.4		−0.2 (2.0)	−0.3; −0.1	
Insomnia									
Total Sample	7.0 (4.7)	6.7; 7.3		11.5 (6.0)	11.2; 11.9		4.5 (5.2)	4.2; 4.8	
Sample COVID Yes	6.8 (4.8)	6.4; 7.3	0.78	12.2 (6.1)	11.6; 12.9	−2.81 **	5.4 (5.8)	4.8; 6.0	−3.77 ***
Sample COVID No	7.1 (4.7)	6.7; 7.4		11.2 (6.0)	10.8; 11.6		4.1 (4.8)	3.8; 4.5	

μ: Average value; σ: Standard deviation: Percentage (x); C.I.: Confidence interval; ***: *p* < 0.001; **: *p* < 0.01; *: *p* < 0.05; ^1^: *Delta* = *Post—pre.*

**Table 5:** Pearson’s correlations between anxiety, hardiness, and insomnia levels in the sample of nurses and in the two groups of nurses caring and not caring for COVID-19 patients.

	Total Sample	COVID Yes	COVID No	Total Sample	COVID Yes	COVID No
Dimension	Insomnia Post			Insomnia Delta		
State anxiety (STAI-Y 2)	0.571 ***	0.539 ***	0.581 ***	0.476 ***	0.511 ***	0.446 ***
Trait anxiety (STAI-Y 1)	0.447 ***	0.464 ***	0.436 ***	0.234 ***	0.284 ***	0.201 ***
Hardiness total (DRS15)	−0.324 ***	−0.290 ***	−0.336 ***	−0.295 ***	−0.341 ***	−0.254 ***
Hardiness comm (DRS15 com)	−0.306 ***	−0.291 ***	−0.309 ***	−0.324 ***	−0.350 ***	−0.301 ***
Hardiness contr (DRS15 con)	−0.195 ***	−0.229 ***	−0.219 ***	−0.226 ***	−0.282 ***	−0.186 ***
Hardiness chall (DRS15 chal)	−0.237 ***	−0.141 **	−0.234 ***	−0.145 ***	−0.201 ***	−0.100 **

***: *p* < 0.001*;* **: *p* < 0.01.

**Table 6:** General linear model ANOVA to assess the best predictive model for the worsening of insomnia levels.

Variable	Between Subjects Test	Parameters	
	*F*	ß	*t* Test
COVID Ward (No)	4.46 *	−0.55	−2.11 *
Anxiety (State)	367.29 ***	0.21	19.16 ***
Commitment (DSR-Delta)	17.13 ***	−0.24	−4.14 ***
Insomnia (Baseline)	225.60 ***	−0.40	−15.02 ***

***: *p* < 0.001*;* *: *p* < 0.05.

## Data Availability

Data supporting reported results are available on request to the corresponding author.

## Public Involvement Statement

No public involvement in any aspect of this research.
